# Reducing Low-Value Care: Uncertainty as Crucial Cross-Cutting Theme: Comment on "Key Factors That Promote Low-Value Care: Views of Experts From the United States, Canada, and the Netherlands"

**DOI:** 10.34172/ijhpm.2022.7027

**Published:** 2022-03-15

**Authors:** Leti van Bodegom-Vos, Perla Marang-van de Mheen

**Affiliations:** Department of Biomedical Data Sciences, Medical Decision Making, Leiden University Medical Centre, Leiden, The Netherlands.

**Keywords:** Low-Value Care, De-Implementation, Medical Overuse

## Abstract

Low-value care is increasingly recognized as a global problem that places strain on healthcare systems and has no quick fix. Verkerk et al have identified key factors promoting low-value care on a national level, proposed strategies to address these and create a healthcare system facilitating delivery of high-value care. In this commentary, we reflect on the results of Verkerk et al and argue that uncertainty has a crucial role when it comes to reducing low-value care. This uncertainty is reflected in lack of a shared view between stakeholders, with clear criteria and thresholds on what constitutes low-value care, and as cross-cutting theme related to the key factors identified. We suggest to work on such a shared view of low-value care and – different from implementation efforts – to explicitly address uncertainty and its driving cognitive biases grounded in human decision-making psychology, to reduce low-value care.

 Low-value care – care that has limited benefit for patients, possibly harms them and stresses limited healthcare resources – is increasingly recognized by policy-makers as a global problem straining healthcare systems. Many have argued that its primary cause lies in an unchecked fee-for-service payment system,^1^ creating a pervasive culture that rewards providers delivering more care rather than the right care. However, the problem is more complex and has no quick fix.^2^ It is therefore important to understand what other factors lead to low-value care and through what mechanisms. Although many studies have identified a wide array of interrelated factors promoting low-value care,^3^ only few studies focused on national-level factors.

 Verkerk and colleagues’ study^4^ in the *International Journal of Health Policy and Management* therefore aimed to identify the national-level key factors promoting low-value care, based on interviews with healthcare professionals, health policy-makers and researchers with experience in identifying and reducing low-value care. The study provides helpful new insights how a mix of national-level system, knowledge and social key factors promote delivery of low-value care. In addition, a number of strategies to reduce low-value care by targeting these key factors are discussed.

 Reflecting on their results, we argue the crucial role of uncertainty when it comes to reducing low-value care. Uncertainty around what constitutes low-value care and uncertainty as cross-cutting theme related to the identified key factors, should be addressed in strategies to reduce low-value care. Managing uncertainty is therefore proposed as a key strategy to reduce low-value care.

## Who Decides What Constitutes Low-Value Care?

 The study by Verkerk et al adds to the wide array of interrelated factors promoting low-value care, although it is striking that no factor related to low-value care itself was identified. The characteristics of low-value care, such as no clear definition of low-value care among all stakeholders, can have an important role in promoting the use of low-value care, just like the characteristics of an innovation (eg, relative advantage, compatibility) within implementation efforts.^5^ We therefore think that an essential part is missing in their exploration of factors promoting low-value care: who decides what constitutes low-value care and according to which criteria?

 Researchers often (implicitly) assume that different stakeholders have the same view and criteria to determine what constitutes low-value care. However, nothing could be further from the truth. Although low-value care concept is well-known, health policy-makers, researchers, patients, the wider public and healthcare professionals do not necessarily have a shared view what it means. Because costs, benefits and harms vary across stakeholders, low-value care can and will have different meaning to different groups.^6^ While healthcare policy-makers may think it is about efficiency of care delivery at population level, healthcare professionals may consider effectiveness or net clinical benefits, and patients and the wider public may only perceive the sum of treatment benefit relative to cost for an individual. Verkerk et al^7^ previously showed that there are several definitions of low-value care, each with different criteria on what gets included, eg, care providing little or no value, care of which the benefits do not outweigh the harms or costs, care that is less effective than alternative care, or care not fitting the patient’s preferences. For the current study, low-value care was defined as care that is proven of little or no value to the patient. But what constitutes little or no value? How do we determine value and does not that differ across stakeholders Until now most initiatives aiming to reduce low-value care, including Choosing Wisely, let healthcare professionals’ organizations decide which tests and treatments have little or no value.^8^ Mostly, they identify commonly used tests and treatments that are not supported by evidence for specific clinical problems, and could expose patients to harm.^9^ This suggests that the effectiveness or net clinical benefit criterium seems to prevail in deciding whether a test or treatment is low-value care.

 Besides different meanings across stakeholder groups, there are also no clear thresholds, eg, to define little or no value. If we were to conduct a clinical trial to assess whether two treatments have similar outcomes (ie, a non-inferiority trial), we need to define the margin between which we consider outcomes as similar. We may need to do the same when defining thresholds for low-value care. What healthcare practitioners think are benefits and acceptable harms for a certain test or treatment, may differ for a patient or health policy-maker. As a result, there is often no agreement when care has little or no value. Agreeing on a uniform margin or threshold, just as we have done for cost-effectiveness analyses, may be an important way forward.

 So, given the influence of multiple stakeholders on provision of low-value care, we need low-value care to become a better defined target, meaning we need to agree upon the criteria on which it should be based as well as uniform thresholds how little or no value is defined. A first step to develop such a shared view, may be consensus building among stakeholders using a Delphi-process.^10^ From this shared view on low-value care, all stakeholders can start working on strategies to reduce the complex low-value care problem.

## Uncertainty as Cross-Cutting Theme Among Factors Promoting Low-Value Care

 Verkerk et al have classified the identified factors promoting low-value care into system (payment structure, industry and malpractice litigation), knowledge (evidence and medical education) and social factors (public culture and medical culture). However, looking in more detail at the description of these factors and sampled quotes per factor, these seem to share uncertainty as cross-cutting theme. Uncertainty refers to situations involving imperfect or unknown information, which diminishes how efficient and effectively we can make clinical decisions, and may result in concerns, fears or anxieties.^11^ Uncertainty is therefore likely underlying the described concerns of healthcare providers to sustain revenue in fee-for-service payment models (payment structure), and fears of healthcare providers to make a mistake and dissatisfy patients, being sued by or get complaints from patients (malpractice litigation). Uncertainties may also play a role in the need for the evidence to be very strong in showing something does not work if it has been done for many years, and medical education focused on beingthorough in ruling out all possible diseases not helpful to accept uncertainty as an inherent part of medicine. Among social factors, uncertainties are related to the poor willingness of patients and society to accept there are always risks and uncertainties, as well as the tendency of healthcare professionals to be ‘better safe than sorry’ trying to rule out such uncertainty.

 All these forms of uncertainty promote low-value care, resulting in more diagnostic testing and treatments that may be avoided if all stakeholders are aware that risk can never be ruled out completely, so that we need to accept that some uncertainty will always remain. The reliance on more diagnostic testing and treatment as a response to uncertainty, is the result of using automatic cognitive processes in decision making under time pressure.^12^ These automatic cognitive processes use mental short cuts (or heuristics) to reduce cognitive load and make decision-making more efficient when the right course of action is not immediately clear. The problem for de-implementation is that these mental short cuts are driven by several cognitive biases, which lead to more diagnostic testing and treatment. Examples of these biases are action bias, referring to people’s preference for action over inaction, and anticipated regret which refers to a strong desire to avoid experiencing regret by not administering a diagnostic test or treatment that could have benefited at least a few recipients, which overpowers any regret for adverse consequences (harms, costs) to patients of whom many will never experience any benefit.

## Managing Uncertainty as a Key Strategy to Reduce Low-Value Care

 Until now, efforts to reduce low-value care, and also most of the strategies proposed by Verkerk et al, can be characterized as directed towards controlled cognitive processes of healthcare providers and the public.^13^ Examples of such strategies are healthcare professional education on harms, providing performance feedback, patient education, and increasing awareness on psychological preconceptions driving low-value care. In these strategies, decision making is expected to change as a function of a conscious intention to change.^13^ However, this assumption largely ignores the dominant role of automatic cognitive processes in clinical decision making, and the fact that reflective processes are ineffective when the underlying uncertainty is not addressed.

 Besides targeting controlled cognitive processes, efforts to reduce low-value care also use circumvention of healthcare providers’ decision making. Verkerk and colleagues’ strategy to move to value-based payment is an example of this, making certain clinical decisions unfavorable. The problem with this type of strategy is that healthcare providers may feel a loss of freedom, potentially resulting in unintended consequences such as increased commitment to certain tests and treatments and finding ways to get them reimbursed.^13^

 Strategies directed at conscious intentions to change and circumventing healthcare providers’ decision making might work for implementation of innovations. However, they seem less effective in de-implementation of low-value care because they do not address uncertainty and the driving cognitive biases that lead to more diagnostic testing and treatments. Several authors have previously argued the importance to address uncertainty in decision making and the driving cognitive biases in de-implementation of low-value care.^12-15^ They propose alternative strategies to reduce low-value care, eg, strategic reframing of non-medical approaches,^12,14^ substitution of a low-value test or treatment,^12-15^ documenting the decision process not to perform a test or treatment^12^ and social support through expert advice and senior review to provide safety netting.^12,15^ In line with these proposed strategies, Patey et al recently showed that de-implementation initiatives more frequently use substitution, monitoring of behavior by others without feedback, and restructuring the social environment (ie, discuss care with other colleagues or obtain signatory authority for a test or treatment) than implementation efforts.^16^ Substituting a low-value test or treatment by an alternative, such as replacing radiography with computerized tomography, is a useful strategy because it provides an alternative action in line with the automatic cognitive processes that healthcare providers use. However, when substitution is not possible, eg, adenotonsillectomy does not have benefits over no treatment in children with mild symptoms of throat infections, then strategic reframing, documenting the decision process and social support may provide alternative strategies.^12^ Not performing an adenotonsillectomy drives against people’s preference for action over inaction (action bias). Educating healthcare providers on harms will not address this action bias, which likely makes education less effective. A more promising strategy might be strategic reframing of the no treatment approach as an active alternative like ‘active monitoring’ to mitigate the impact of action bias in treatment decisions. Not performing an adenotonsillectomy can also lead to fears among healthcare providers for patient complaints and malpractice litigation. Verkerk et al propose to reduce these fears by protecting clinicians from the burden of a complaint, but do not describe how we should accomplish this. Enabling and encouraging healthcare providers to document the decision process resulting in not performing an adenotonsillectomy, including documentation of patient involvement in discussions about values and goals of care, or social support through expert advice and senior review, could be effective strategies. These strategies protect healthcare providers from accusations of carelessness or neglect and can help them feel safer and tolerate their fears for patient complaints and malpractice litigation. Although these suggested strategies to mitigate uncertainty and the driving cognitive biases seem promising, future research should reveal whether they are effective in tackling the global problem of low-value care.

## Conclusion

 Uncertainty plays a crucial role in efforts to reduce low-value care. Health policy-makers may have an important facilitating role in working towards a shared view on what constitutes low-value care, with clear criteria and thresholds. Furthermore, future national-level strategies should address the cross-cutting theme of uncertainty across factors promoting low-value care to make these strategies more effective. Until now, de-implementation strategies rely too frequently on insights obtained from implementation. However, because of uncertainty and its driving cognitive biases, de-implementation asks for strategies that take into account the pervasive asymmetry in human decision making.

## Ethical issues

 Not applicable.

## Competing interests

 Authors declare that they have no competing interests.

## Authors’ contributions

 LvBV and PMvM co-created the manuscript. Both read and agreed the final version.
